# First Test of a Potential Biological Control Agent of Argentine ants (*Linepithema humile*)

**DOI:** 10.3390/insects16070677

**Published:** 2025-06-28

**Authors:** Patricia J. Folgarait, Daniela Goffré

**Affiliations:** Ant Ecology and Control Laboratory, Department of Science and Technology, National University of Quilmes, CONICET, Bernal B1876BXD, Buenos Aires, Argentina; daniela.goffre@unq.edu.ar

**Keywords:** *Beauveria bassiana*, entomopathogen, fungal pathogen, invasive ants, native range

## Abstract

The Argentine ant (*Linepithema humile*), listed among the world’s 100 worst invasive alien species, is notoriously difficult to control due mainly to its formation of large, expansive supercolonies. Despite the drawbacks of chemical control, biological alternatives have not been previously explored for this species. In this study, we evaluated six native entomopathogenic fungal strains against Argentine ants from four behaviorally distinct supercolonies, identified through aggression assays and collected from both urban and natural sites within the species’ native range. Ants were inoculated with 1 × 10^8^ conidia/mL using three methods: topical application, spray, and immersion. Mortality was recorded over 14 days, and the cause of death was confirmed by fungal outgrowth from cadavers. Among all strains, *Beauveria bassiana* Li053 consistently induced high mortality across all supercolonies and inoculation methods, with LT_50_ values between 2 and 5 days and final mortality rates exceeding 80%. Fungal infection was confirmed in 87–92% of cadavers. Dose–response assays revealed that higher conidial concentrations accelerated and increased mortality, with an LC50 estimated at 1 × 10^6^ conidia/mL. These results demonstrate that *B. bassiana* Li053 is a promising candidate for the biological control of *L. humile* and merits further evaluation under field conditions.

## Simple Summary

Argentine ants are a major invasive species worldwide, forming massive supercolonies that threaten native wildlife, damage crops indirectly by protecting sap-feeding insects, and harm beehives and irrigation systems. Traditional control efforts using synthetic chemicals have had limited success and are unsustainable. Biological control—using natural enemies—offers a safer alternative, but has never been tested against this species until now. We isolated six strains of entomopathogenic fungi from Argentine ants in their native range and tested them in the laboratory on ants from four distinct native colonies. One strain, *Beauveria bassiana* Li053, more consistently caused over 80% mortality, killing half the ants within 2–5 days. Higher spore concentrations led to quicker and greater final mortality. These findings show strong potential for biological control of Argentine ants. Such a method is environmentally friendly, more specific to the target species, safer for beneficial insects and humans, and sustainable over time.

## 1. Introduction

Invasive ants in non-native environments pose significant threats to local biodiversity, affecting a wide range of taxa from invertebrates to vertebrates, often causing negative ecological and socio-economic impacts [1,2]. Chemical control has traditionally been the primary strategy for managing invasive ant populations. However, these methods were effective only at local scales and typically required repeated applications, and reinvasion is frequently observed even in areas where eradication was initially successful [3,4]. Repeated use of synthetic insecticides has raised persistent concerns due to their documented negative effects on human health, non-target organisms, and the environment (i.e., [5,6]). Despite the urgent need for safer and more sustainable alternatives, only a few methods have been proposed and preliminarily tested against invasive ants in non-native areas, including the application of hot water [7], biological control agents [8], natural compounds and materials [9], semiochemicals [10], and physical or cultural practices [11]. However, these approaches have not yet been commercialized or widely adopted in routine management programs.

The Argentine ant (*Linepithema humile* (Mayr, 1868)), native to the floodplains of the Paraná and Paraguay rivers [12], is a well-known invasive species in the United States, Europe, New Zealand, Australia, and Korea [12,13,14,15,16,17,18,19] and has been recorded in more than 50 countries [20]. Its introduction into non-native ecosystems has led to significant declines in native ant diversity [21] and broader arthropod communities [22,23,24], triggering trophic cascades that threaten ecological stability [25]. As such, it is one of the only five ant species included in the list of the 100 worst invasive alien species worldwide [26].

Its invasive success is largely attributed to the formation of expansive supercolonies characterized by interconnected nests (unicoloniality) and an absence of intraspecific aggression [27]. *L. humile* is also polygynous and highly aggressive toward other species, facilitating rapid territorial expansion [28]. The most notable example is a 6000 km-long supercolony spanning Spain, Portugal, and northern Italy, established over a century ago [16]. Beyond its impacts on biodiversity, *L. humile* poses a nuisance in urban areas and households in both its native and invasive ranges [29]. It also causes substantial agricultural damage by tending and protecting phloem-feeding pests (e.g., aphids), attacking beehives, eating the juveniles and forcing the bees to abandon their nests, and disrupting irrigation systems by chewing drip tube walls and enlarging their holes [30,31].

Control efforts using synthetic insecticides have achieved limited and localized success against Argentine ants and often require repeated applications [25,32,33]. Alternative strategies under investigation include synthetic pheromones, behavior-modifying chemicals, bait traps, hydrogels with low insecticide concentrations, plant-based repellents, and RNA interference (RNAi), with the latter two still in early stages of development [34]. Environmental modifications, such as providing external water sources to reduce structural infestation, have also been explored [29,35].

To date, no biological control agents have been tested for *L. humile*, and few natural enemies have been studied. Although viruses have been detected in association with this species [36], their biocontrol potential remains untested. Known *Linepithema* parasitoids do not target *L. humile* [37], and repeated surveys have failed to identify any for this species (Folgarait, pers. obs.), limiting the applicability of this approach [38,39]. One study reported the isolation of three fungal species from Argentine ants; however, the study was not designed to evaluate their potential for biological control [40]. While *L. humile*’s chemical defenses have some antibiotic activity [40,41], their relevance to control strategies remains unclear. Although the Argentine ant is one of the most extensively studied ant species, with over 1000 scientific publications since the last century [20], it is striking that no published studies have explored the biological control of this species. Our study addresses this critical knowledge gap and proposes a native fungal strain as a promising candidate for the biological control of *L. humile.*

Despite the complex hygienic behaviors of social insects [42], pathogenic fungi have shown promise against other ant species when formulated as baits [43,44,45], suggesting potential for future application against *L. humile*. Therefore, the objective of this study was to isolate and identify entomopathogenic fungal strains naturally associated with native Argentine ants and to assess, for the first time, their potential as biological control agents. We evaluated the virulence of these isolates by experimentally inoculating *L. humile* workers using three different methods, and monitoring both mortality and fungal recovery from ant cadavers. The most virulent fungal strain and the most effective inoculation method were then used to determine the median lethal concentration (LC50) by exposing ants to increasing conidial concentrations. Prior to the bioassays, aggression tests were conducted to determine colony boundaries and confirm whether ants originated from distinct colonies, ensuring an appropriate experimental design.

## 2. Materials and Methods

### 2.1. Ant Collection

Ants were collected from four sites within Buenos Aires Province: two located in natural reserves and two in urban places. Distances between sites ranged from 12.9 to 47.6 km. In the reserves, several transects were established with 10 bait stations each, separated by 10 m. From the 20 transects placed at the Ecological Reserve Costanera Sur (RECS), only 7 did not have *L. humile*. At the Integral and Mixed Natural Reserve “Laguna de Rocha”, we were only able to place 10 transects because there is less open space. From those, we found Argentine ants in 50% of them. In the urban sites, ants were collected from 2–3 focal points of the foraging trail on different days/months. Baits consisted of a piece of sausage, sometimes complemented with a cotton ball soaked in sugar water solution. All Argentine ants attracted to the bait were collected and placed in a plastic container (23 × 12 × 6 cm) with Fluon-coated walls to prevent ant escape. Baits were replaced, as ants were collected, until either the required number of ants was obtained (~800) or foraging activity ceased, whichever occurred first. Inside the containers, sausage or crickets, along with a 20% sugar solution, plus water, were provided ad libitum. The ants were taken to the laboratory the next day after collection. Ants were collected between 2021 and 2024. In 2021, ants were collected for natural fungal isolations and to perform aggression tests, while in 2023 and 2024, samples were used for virulence experiments and to evaluate the effect of time on aggression tests. Therefore, we collected ants opportunistically and performed new aggression tests in the morning (9:00 to 12:00), noon-early afternoon (12:00 to 17:00), and late afternoon (after 17:00).

### 2.2. Aggression

Aggression tests were performed in the laboratory using ants that had, at most, 1 week after they were collected in the field. Each aggression test had 1 experimental treatment where 5 ants from two stations/transects, from the same or different sites, were placed simultaneously and together in a neutral vial, and their interaction was observed during the first minute, and afterwards for 5 more minutes. Interactions were scored from 0 to 4, indicating an increasing level of aggression. Scores 3 and 4, with the greatest level of aggression, indicated that the confronted ants belonged to different colonies. Scores 0 and 1, with the lowest aggression, indicated that the pair of tested ants belonged to the same colony. Score 2, which refers to ants avoiding each other, was recorded, but it was the most ambiguous score and was not included in the analyses (see [46] for score categories). In addition, each aggression test also had two controls, each representing individuals from the confronted pair. Controls were conducted by placing 10 ants from the same origin in the same vial; these were observed and scores recorded as explained earlier. The highest score recorded in each aggression test was used for analysis. The tests were conducted blindly: one person assigned a number to each vial—replacing the ant source—and the other person recorded the behaviors and assigned scores.

### 2.3. Isolation and Identification of Fungal Strains

Dead ants collected from the two natural reserves were individually placed in humid chambers and periodically observed under a binocular microscope for signs of fungal growth emerging from within the cadavers. Fungi growing from the ant cadavers were collected from their surfaces and cultured on potato dextrose agar (PDA) in Petri dishes. Fungal cultures were subcultured weekly onto fresh PDA plates until pure cultures were obtained. After that, fungi were stored in glycerol 20% in a −80 °C freezer. In order to identify the fungal isolates, first, we classified the fungi in morphotypes. At that time, the 6 isolated strains received the following identification codes: Li003, Li015, Li053, Li063, Li124, and LiS/ID.

Second, and in order to determine the species identity, we performed molecular identifications by extracting genomic DNA from mycelia, following a modified version of the cetyltrimethylammonium bromide (CTAB) method [47]. Mycelium from each strain was obtained from a fresh culture (3 days on PDA) and ground in a frozen mortar with 500 μL of 2% CTAB buffer and 1 μL of β-mercaptoethanol. Exceptions were made for *Aspergillus* Li015, where 8 μL of β-mercaptoethanol was added, and for *Aspergillus* Li003, where 4 μL of β-mercaptoethanol and 1 μL of proteinase K (20 mg/mL) (Sigma-Aldrich, St. Louis, MO, USA) were used. The resulting mixture was transferred to a 1.5 mL tube and briefly centrifuged at 14,000 rpm. Afterwards, 500 μL of chloroform was added, the tube was vortexed, and centrifuged again at 14,000 rpm for 15 min. The aqueous phase was transferred to a new 1.5 mL tube. These steps were repeated three times for both *Aspergillus* Li003 and Li015. Then, one volume of cold pure isopropanol was added, and the samples were incubated for 1 h at −80 °C to precipitate the DNA. After that time, the samples were centrifuged at 14,000 rpm for 15 min, and the resulting pellet was washed with 75 μL of 70% ethanol and centrifuged for 5 min. The supernatant was discarded, the pellet was air-dried for 1 min, and then resuspended in 50 μL of TE buffer (10 mM Tris-HCl, pH 7.6–8.0; 0.01 mM EDTA) diluted 1:10. DNA integrity was assessed with agarose gel electrophoresis using GelRed (Biotium, Fremont, CA, USA) for DNA visualization on an ultraviolet (UV) transilluminator, and DNA concentration was estimated by absorbance at 260 nm using a Nanodrop spectrophotometer (Thermo Fisher Scientific, Waltham, MA, USA).

After that, we amplified an approximately 600 bp fragment of the nuclear 28S ribosomal DNA (28S rDNA), using the primers LR0R [48] and LR5 [49]. The polymerase chain reaction (PCR), reaction mixture, and the thermocycling protocol were the same as those described in [50]. PCR products were analyzed using agarose gel electrophoresis using GelRed for DNA visualization as previously described. Amplified products were sent to Macrogen (Seoul, Republic of Korea) for bidirectional sequencing. The sequences obtained with each primer were assembled and cleaned using MEGA11 [51], and the resulting consensus sequences were compared to known sequences in GenBank (National Center for Biotechnology Information, U.S. National Library of Medicine, Bethesda, MD, USA), using the nucleotide–nucleotide BLAST algorithm (https://blast.ncbi.nlm.nih.gov/Blast.cgi, accessed 24 March 2025). The identification of each fungal strain was based on BLAST searches of the amplified 28S rDNA sequences from GenBank.

For strains that could not be resolved to species level based only on nuc 28S rDNA sequences, morphological characteristics were used to complement molecular identification. We examined colony color, texture, and growth patterns on PDA, as well as conidial morphology under the microscope, and compared them with published descriptions of reference species.

### 2.4. Inoculation Methods and Virulence Tests

We employed three inoculation methods to assess the pathogenicity of six different fungal strains, as these represent approaches used in the literature [45]. The topical method, involving the direct application of a drop of suspension onto the ant’s body, is the least commonly used. The spray method, where ants were exposed to a known volume of suspension via spraying, was selected for its practical relevance. This technique simulates potential field applications, such as treatment using a backpack sprayer. Finally, the immersion method, in which ants were submerged in a conidial suspension for a defined period, is the most frequently used technique in ant pathogenicity studies.

Each fungus was cultured on PDA in Petri dishes for 7–8 days in a laboratory incubator (San Jor, San Martín, Argentina) at 25 °C in complete darkness. Afterwards, conidia were harvested from the surface of the cultures using a 0.01% Tween 80 solution, to prepare suspensions at a final concentration of 1 × 10^8^ conidia/mL, except for Li063 which was adjusted to 5 × 10^7^ conidia/mL. Conidia concentrations were determined using a Neubauer hemocytometer and adjusted through serial dilutions to reach the required concentration.

To assess the virulence of each fungal strain, we used 100 ants for each inoculation technique and collection site. In the topical technique, 1 µL drop of the conidial suspension was pipetted onto the thorax of each ant. For the spray method, the suspension was sprayed twice (approximately 100 µL per spray), directly into the flask containing the ants. For the immersion technique, 5 mL of fungal suspension was added to a flask where the ants had previously been added, and the suspension was gently agitated for 30 s to ensure full submersion. The liquid was then removed. For all three inoculation methods, control treatments followed the same procedure but used a 0.01% Tween 80 solution instead of the fungal suspension. After inoculations, both experimental and control groups were maintained in containers supplied with a piece of paper towel and a tube with 20% sugar solution. The following day, only those ants that had survived the inoculation procedure (mortality never exceeded 10%) were transferred to a new container with their sugar and water tubes. Dead ants were counted, removed, and individually transferred to sterile humid chambers every other day.

To determine the cause of death, each ant cadaver was subsequently examined under a microscope to assess whether fungal growth emerged from within the body, particularly at the joints and intersegmental regions. When present, the fungi were identified as either the inoculated strain or a different species. Ants were not surface-sterilized, as the objective was to evaluate the virulence of each inoculated fungal strain under conditions in which ants retained their naturally occurring pathogen load.

### 2.5. Median Lethal Concentration Assays

With the strain that exhibited the highest virulence, we calculated the median lethal concentration (LC50) by evaluating ant mortality using different concentrations, as follows: 1 × 10^5^, 1 × 10^6^, 1 × 10^7^, and 1 × 10^8^ conidia/mL. Groups of 100 ants from each of the four sites were submerged separately using the immersion technique described previously. Control ants were immersed in the same volume for the same amount of time (30 s) but in a 0.01% Tween 80 solution. Afterwards, we checked for mortality using the same protocol as described above.

### 2.6. Statistical Analyses

Among all the results obtained from the aggression tests, we compared the frequency of scores between the least aggressive behaviors (scores 0 and 1) and the most aggressive ones (scores 3 and 4) across different sites or the same one (controls). A Chi-square test was used to assess if there was any association between the origin of the ant pairs and the scores obtained during confrontations.

Most of the results from the virulence experiments consisted of lifetime data and, therefore, were analyzed using the non-parametric Kaplan–Meier test, which estimates the survival function. We used the Mantel test to obtain a *p*-value, which was adjusted for multiple comparisons using the Bonferroni correction. We also calculated the time required to reach 50% ant mortality (LT50). The raw survival data used for statistical analyses are available in Supplementary Table S1.

To estimate the LC50 and LC90, we performed a Probit analysis [52]. Cumulative mortalities on the fifth day (the day on which mortalities were still below 100%) were corrected using Abbott’s formula: (% treatment mortality − % control mortality)/(100 − % control mortality) [53].

All analyses were conducted using Systat software, version 13 [54].

## 3. Results

### 3.1. Aggression

Aggression tests conducted within sites revealed no aggressive behavior in any of the 30 tests performed per natural reserve. Similarly, at the urban sites, no aggression was observed in any of the 12 tests at Bernal or the 7 at Gonnet, with a single exception recorded at the Gonnet site. In contrast, confrontations between ants from different sites yielded aggression scores of 3 or 4 in 23 out of 27 tests.

Of the 199 aggression tests, 28% were conducted in the morning, 37% at midday to early afternoon, and 35% in the late afternoon. During morning trials, 21 tests received scores of 0 or 1, 19 of which corresponded to control ants (ants from the same site), whereas the remaining 35 tests, which received scores of 3 or 4, involved ants from different sites. Among the tests performed from noon to early afternoon, 43 had scores of 0 or 1, with 37 corresponding to control ants; the remaining 31 tests, which scored 3 or 4, involved ants from different sites. In the late afternoon, 30 tests scored 0 or 1, 26 of which involved control ants, while 38 out of the remaining 39 tests involving ants from different sites received scores of 3 or 4. Therefore, the frequency of “mistakes”, or deviations from the main pattern found, did not appear to vary with the time of day at which the aggression tests were conducted (Table 1).

Combining results across all time periods, 93 observations yielded scores of 0 or 1, while 106 had scores of 3 or 4 (Table 1). The low (0 + 1) and high (3 + 4) aggression scores were dependent on the pair of ants tested (χ^2^ = 162.5, df = 9, *p* < 0.001). When only control group data were considered, the low and high aggression scores were independent of the pair of sites compared (χ^2^ = 3.14, df = 3, *p* = 0.37). The same was observed when the aggression tests were conducted with ants from different sites (χ^2^ = 13.13, df = 5, *p* = 0.02, but non-significant after Bonferroni correction). Due to the concentration of results in one of the two score categories, several cells in the latter two comparisons did not meet the expected value assumptions for the chi-square test.

### 3.2. Isolation and Identification of Fungal Strains

All the samples were originally isolated from the Rocha Reserve, except for Li003, which was collected from the RECS reserve (Table 2).

The species from reference collections showing the highest identity scores based on 28S rDNA BLAST results, along with the final species identification, are summarized in Table 2.

Li053 showed 99.68% identity only to *Beauveria bassiana* collection strains ATCC 26854 and CBS:212.61. Strain Li003 matched *Aspergillus caelatus* CBS 763.97 with 99.75% identity, while strain Li015 showed a lower identity (94.84%) to the same species. In contrast, strains LiS/ID, Li124, and Li063 could not be resolved to the species level based solely on 28S rDNA data because their sequences matched with more than one reference species. LiS/ID showed a match with the reference strains of the species *Metarhizium* *anisopliae* (CBS:662.67) and *M. album* (ARSEF:2082), both belonging to the *M. anisopliae* species complex [55]. Similarly, Li124 matched *Purpureocillium lilacinum* and *P. lavendulum* with 100% identity to CBS:129410 and CBS:128678, respectively, and with 99.29% to another reference strain of *P. lilacinum* (ATCC 10114). Strain Li063 presented 100% identity with several *Fusarium* species, including *F. oxysporum*, *F. incarnatum,* and *F. redolens*, among others; therefore, this strain could not be unequivocally assigned to a single *Fusarium* species.

Due to the lack of resolution for some of the strains, we complemented molecular identification with morphological observations. In the case of Li124, its morphology was consistent with the typical *P. lilacinum* [56], and LiS/ID matched that of *M. album* [57]. Likewise, strain Li063 presented colony morphology and conidial characteristics compatible with *F. oxysporum* [58] (Table 2). We also confirmed morphologically those strains that were identified molecularly at the species level (strains Li053, Li003, and Li015) and confirmed that their morphological features matched those reported for the assigned species [59,60].

### 3.3. Virulence Tests: Survival and Cause of Death by Colony

#### 3.3.1. Urban Site: Bernal

**Figure 1:**
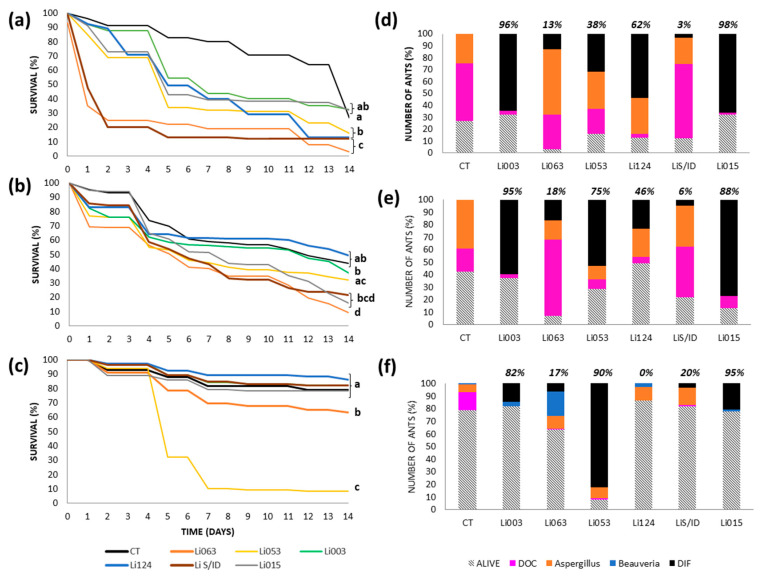
Survival and fungal recovery of *Linepithema humile* ants from the Bernal colony inoculated with different fungal strains using three inoculation methods. (**a**–**c**) Survival curves of ants treated with six fungal strains (Li003, Li015, Li053, Li063, Li124, LiS/ID) and their respective controls (CT), using topical application (**a**), spray (**b**), or immersion (**c**). Different letters indicate statistically significant differences between treatments (*p* < 0.002). (**d**–**f**) Proportion of surviving ants (bars with diagonal lines) and causes of death corresponding to each inoculation method: topical (**d**), spray (**e**), and immersion (**f**). Colored bars represent the percentage of ants that died due to inoculated fungal strain (black bar, DIF), as well as the proportion of cadavers with recovery of naturally occurring *Aspergillus* (orange bar) and *Beauveria* (blue bar), and those with unidentified causes of death (fuchsia bar, DOC). The proportion of ants from which the inoculated fungal strain was recovered, calculated relative to the total number of cadavers, is indicated at the top of each bar.

Using the topical method, ant survival was significantly lower than in the control treatment (*p* < 0.002) for four of the six fungal strains tested (Li053, Li124, Li063, and LiS/ID), resulting in 80–90% mortality by the end of the experiment (Figure 1a). Among these, Li063 and LiS/ID caused faster mortality than Li053 and Li124, with LT50 values of 1 day for both strains, compared to 4–5 days for the latter two (Figure 1a). However, strain recovery varied and seemed not to be related to the mortality observed. Li063 and LiS/ID were recovered from only 13% and 3% of total ants, respectively, as well as from dead ants (black bars in Figure 1d). In contrast, Li124 and Li053 were recovered from 54% and 32% of all ants, or from 62% and 38% of the ant cadavers, respectively. For Li063, the majority of ant deaths (55%) were caused by a naturally occurring *Aspergillus* strain, which was also isolated from 25% of control ants. In the case of LiS/ID, most deaths (62%) were due to unidentified causes unrelated to fungal infection (Figure 1d).

With the spray method, strains Li063 and LiS/ID again caused significantly lower survival of ants compared to controls, along with an additional strain, Li015; all three resulted in 80–90% mortality (Figure 1b). For these strains, the LT50 ranged from 6 to 8 days, indicating slower mortality than with the topical application. Strain recovery remained low for Li063 and LiS/ID, detected in 17% and 5% of total ants, or 18% and 6% of dead ants, respectively. In contrast, Li015 was recovered from 77% of all the ants, accounting for 89% of cadavers (Figure 1e). For Li063, 61% of mortality was attributed to unidentified causes, whereas for LiS/D, 41% of deaths were due to unknown causes, and 32% were associated with the naturally occurring *Aspergillus* strain, similar to the 39% observed in the control group.

In contrast, the immersion method yielded markedly different results. Strain Li053 caused significantly greater mortality (89%) compared to all other treatments (Figure 1c), with an LT50 of 5 days. Recovery of Li053 was also high, found in 83% of total ants or 90% of cadavers (Figure 1f). Control group mortality was minimal and mainly associated with other causes (67% of cadavers) and a low prevalence of naturally occurring *Beauveria* (4%) and *Aspergillus* (29%) strains, as well as deaths attributed to unknown causes (Figure 1f).

#### 3.3.2. Urban Site: Gonnet

**Figure 2:**
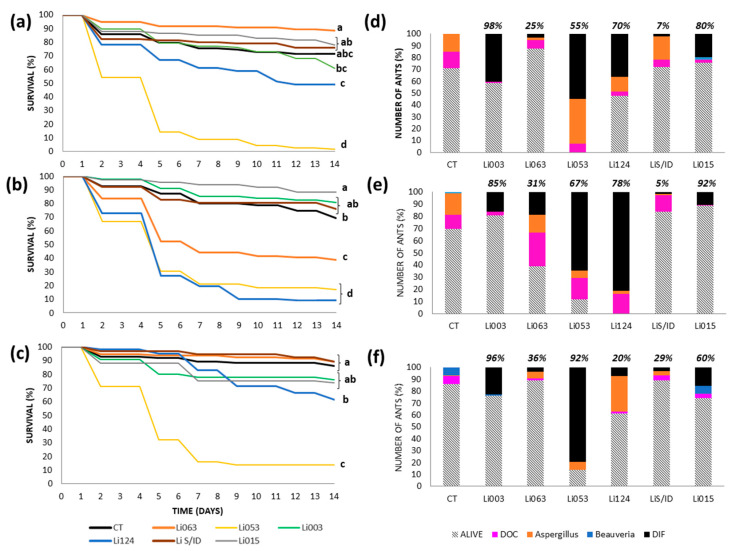
Survival and fungal recovery of *Linepithema humile* ants from the Gonnet colony inoculated with different fungal strains using three inoculation methods. (**a**–**c**) Survival curves of ants treated with six fungal strains (Li003, Li015, Li053, Li063, Li124, LiS/ID) and their respective controls (CT), using topical application (**a**), spray (**b**), or immersion (**c**). Different letters indicate statistically significant differences between treatments (*p* < 0.002). (**d**–**f**) Proportion of surviving ants (bars with diagonal lines) and causes of death corresponding to each inoculation method: topical (**d**), spray (**e**), and immersion (**f**). Colored bars represent the percentage of ants that died due to inoculated fungal strain (black bar, DIF), as well as the proportion of cadavers with recovery of naturally occurring *Aspergillus* (orange bar) and *Beauveria* (blue bar), and those with unidentified causes of death (fuchsia bar, DOC). The proportion of ants from which the inoculated fungal strain was recovered, calculated relative to the total number of cadavers, is indicated at the top of each bar.

Using the topical method, ant survival was the lowest with strain Li053, resulting in 100% mortality and an LT50 of 5 days (Figure 2a). This strain was recovered from 55% of total ants, as well as from ant cadavers, while 38% of the ants were infected with a naturally occurring *Aspergillus* strain (Figure 2d). Li124Y also caused significantly higher mortality than the control; however, it only killed 52% of the ants, and was recovered from only 36% of all the ants, or 70% of ant cadavers.

Using the spray method, strains Li053 and Li124Y again caused the greatest mortality, resulting in 80–90% of the dead ants (Figure 2b). The LT50 was 5 days for both strains. Li053 was recovered from 65% of total ants or 67% of cadavers, while Li124Y was detected in 81% of all ants, which represented 78% of dead ants (Figure 2e). Li063 also produced significantly lower survivorship than the control, but with a very low recovery, just 19% from all ants, representing 31% of ant cadavers.

With the immersion method, the greatest mortality (86%) corresponded to Li053, which was recovered from 79% of the total number of ants, or from 92% of ant cadavers (Figure 2c,f), with an LT50 of 5 days. In addition, Li124Y also produced significantly greater mortality than the control group, but ended with low mortality (39%); therefore, we were not able to calculate the LT50.

For all three methods, control ants exhibited low mortality rates, ranging from 14 to 31% (Figure 2a–c). Deaths in topical and sprayed ants were primarily attributed to a naturally occurring *Aspergillus* strain and other unknown causes, which together accounted for more than 90% of total deaths. Under immersion, half of the cadavers showed *Beauveria* or other causes (Figure 2d–f).

#### 3.3.3. Reserves: RECS

**Figure 3:**
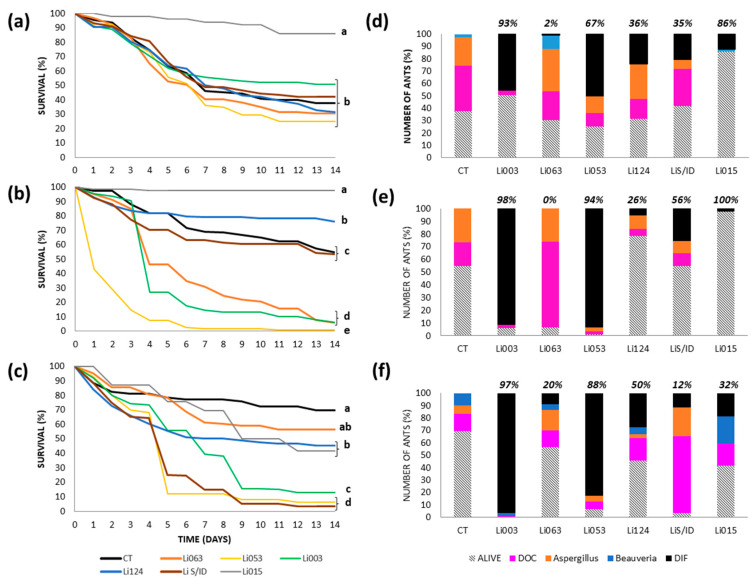
Survival and fungal recovery of *Linepithema humile* ants from the RECS colony inoculated with different fungal strains using three inoculation methods. (**a**–**c**) Survival curves of ants treated with six fungal strains (Li003, Li015, Li053, Li063, Li124, LiS/ID) and their respective controls (CT), using topical application (**a**), spray (**b**), or immersion (**c**). Different letters indicate statistically significant differences between treatments (*p* < 0.002). (**d**–**f**) Proportion of surviving ants (bars with diagonal lines) and causes of death corresponding to each inoculation method: topical (**d**), spray (**e**), and immersion (**f**). Colored bars represent the percentage of ants that died due to inoculated fungal strain (black bar, DIF), as well as the proportion of cadavers with recovery of naturally occurring *Aspergillus* (orange bar) and *Beauveria* (blue bar), and those with unidentified causes of death (fuchsia bar, DOC). The proportion of ants from which the inoculated fungal strain was recovered, calculated relative to the total number of cadavers, is indicated at the top of each bar.

Ant mortality following topical application was very similar across most fungal strains, except for Li015, which caused negligible mortality (Figure 3a). Li053 caused the greatest mortality (75% by the end of the experiment), although this was not significantly different from that caused by most of the other strains. This fungus was recovered from 50% of all ants or 67% of the cadavers (Figure 3d), with an LT50 of 7 days. In the control treatment, 62% of the ants died, due to unknown reasons (59%) or infections caused by naturally occurring *Aspergillus* (36%) and *Beauveria* (5%) strains.

Under the spray method, Li053 caused the highest mortality (99%), and was recovered from 93% of all ants and 94% of dead ants (Figure 3b). Two other strains, Li003 and Li063, each resulted in 94% mortality. However, while Li003 was recovered from 91% of total ants and 98% of cadavers, the other strain was not recovered from any ant (Figure 3e). LT50 values were 1 day for Li053 and 4 days for both Li003 and Li063. In the control group, 45% of all ants died (Figure 3b), with 59% of deaths attributed to a naturally occurring *Aspergillus* strain and 42% to unknown causes (Figure 3e).

The greatest mortality (greater than 90%) recorded with the immersion method was caused by Li053 and LiS/D (Figure 3c), both with LT50 values of 5 days. However, strain recovery varied differently from mortality: LiS/ID was recovered from only 11% of all ants or 20% of cadavers, whereas Li053 was recovered from 83% of all ants and 88% of the cadavers (Figure 3f). Li003 also produced high mortality (87%) and was strongly associated with the observed deaths, as it was recovered from 97% of cadavers (Figure 3f), but mortality progression was slower, with an LT50 of 7 days. In addition, strains Li015 and Li124 produced significantly greater mortality than the control ants, although final mortality was around 55%. The LT50 values were 9 days and 7 days, respectively, and the recovery of these strains from ant cadavers was 50% or lower. The control group showed lower mortality under the immersion method compared to the other two methods, with a 30% of the ants dead by the end of the experiment, mainly due to unknown causes (46%) and *Beauveria* (33%), along with a 21% of deaths attributed to a naturally occurring *Aspergillus*.

#### 3.3.4. Reserve: Rocha

**Figure 4:**
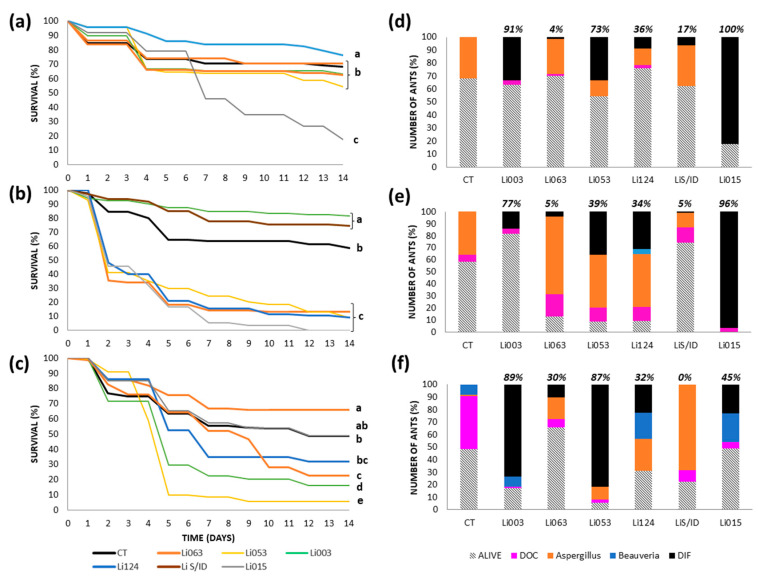
Survival and fungal recovery of *Linepithema humile* ants from the Rocha colony inoculated with different fungal strains using three inoculation methods. (**a**–**c**) Survival curves of ants treated with six fungal strains (Li003, Li015, Li053, Li063, Li124, LiS/ID) and their respective controls (CT), using topical application (**a**), spray (**b**), or immersion (**c**). Different letters indicate statistically significant differences between treatments (*p* < 0.002). (**d**–**f**) Proportion of surviving ants (bars with diagonal lines) and causes of death corresponding to each inoculation method: topical (**d**), spray (**e**), and immersion (**f**). Colored bars represent the percentage of ants that died due to inoculated fungal strain (black bar, DIF), as well as the proportion of cadavers with recovery of naturally occurring *Aspergillus* (orange bar) and *Beauveria* (blue bar), and those with unidentified causes of death (fuchsia bar, DOC). The proportion of ants from which the inoculated fungal strain was recovered, calculated relative to the total number of cadavers, is indicated at the top of each bar.

Under the topical method, Li015 caused the highest mortality (87%), and it was the only strain significantly different from the other treatments, with an LT50 of 7 days (Figure 4a). This fungus was recovered from 83% of all ants, representing the only cause of death among the cadavers (Figure 4d). In the control group, only 32% of the ants died, and all deaths were attributed to a naturally occurring *Aspergillus* strain.

When the inoculation was conducted using the spray method, 4 strains (Li015, Li053, Li063, and Li124) exhibited significantly greater mortality (>86%) than the control, all showing the same LT50 value of 2 days (Figure 4b). However, only Li015 caused 100% of mortality by the end of the assay. Again, mortality was not always associated with recovery percentages: Li015 was recovered from most of the ant cadavers (96%), while Li053 and Li124 were found in approximately 30% of total ants in each case (or 39% and 34% of cadavers, respectively) (Figure 4e). For both latter strains, 44% of ant cadavers showed a naturally occurring *Aspergillus* strain. Li063 was recovered from only 4% of the ants (or 5% of cadavers), and again, the naturally occurring *Aspergillus* strain was the cause of death for 74% of ant cadavers. In the control treatment, 41% of ants died, with 86% of the ant cadavers attributed to a naturally occurring *Aspergillus*.

Under the immersion method, Li053 and Li003 produced significantly greater mortality than the control treatment, both with LT50 values of 5 days. Li053 caused 94% mortality and Li003 an 83% (Figure 4c). These strains accounted for most of the mortality recorded, explaining 87% of deaths in the Li053 treatment and 89% in the Li003 group. In the control treatment, 52% of ants died, with 82% of deaths attributed to unknown causes and 16% due to a naturally occurring *Beauveria* strain (Figure 4f).

### 3.4. Median Lethal Concentration

**Figure 5:**
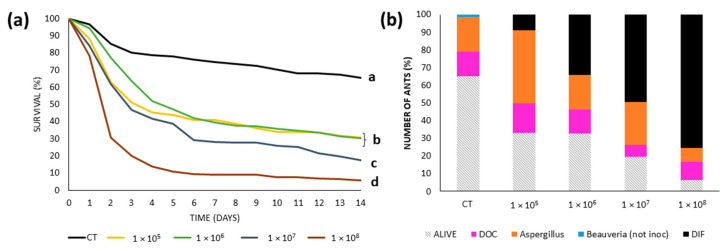
Average survival and fungal recovery of *Linepithema humile* ants from the 4 colonies inoculated with increasing concentrations of *Beauveria bassiana* Li053 using the immersion technique (**a**) Survival curves of ants treated with different concentration (1 × 10^5^–1 × 10^8^ conidia/mL) and control (CT). Different letters indicate statistically significant differences between treatments (*p* < 0.005). (**b**) Proportion of surviving ants (bars with diagonal lines) and causes of death corresponding to each concentration. Colored bars represent the percentage of ants that died from all ants discriminated by the proportion of dead ants recovered with the inoculated fungal strain (black bar, DIF), the proportion of cadavers with recovery of naturally occurring *Aspergillus* (orange bar) and *Beauveria* (blue bar), as well as those with unidentified causes of death (fuchsia bar, DOC).

Overall, the higher the concentration in the conidia solution, the greater the mortality registered. Ants inoculated with 1 × 10^8^ conidia/mL showed greater mortality than those ants treated with lower concentrations, and all fungal treatments generated more mortality than the control group (Figure 5a). The LC50 was 1.2 × 10^6^ conidia/mL, and the LC90 was 2.1 × 10^9^ conidia/mL.

The proportion of dead ants from which *Beauveria bassiana* strain Li053 was recovered (Figure 5b) increased with inoculum concentration, from 13% at 1 × 10^5^ conidia/mL to 81% at 1 × 10^8^ conidia/mL. Intermediate recovery rates were observed at 1 × 10^6^ (51%) and 1 × 10^7^ (62%) conidia/mL. In the control group, 35% of all the ants died, of which the majority (56%) and minority (4%) were explained by a naturally occurring *Aspergillus* or *Beauveria* strain, respectively.

## 4. Discussion

### 4.1. Aggression

The aggression results support the presence of unicoloniality in *L. humile* within its native range, as most control tests showed no aggression. At both natural reserves, ants were collected from multiple locations without exhibiting within-reserve aggression, confirming the formation of a supercolony at each site. This finding aligns with previous evidence of unicoloniality in the native range [61,62]. In contrast, aggressive interactions—reflected in high aggression scores—were consistently observed between ants from different sampling sites, suggesting that each site represented a distinct colony. Similar intercolony aggression has been reported within regions [46] and between regions [63,64].

To our knowledge, this study represents the first systematic evaluation of diel variation in aggressive behavior in ants. We found no significant differences in aggression levels across time periods. Only a few exceptions—or “mistakes”—were recorded, where low aggression scores occurred in tests between ants from different colonies, contrary to expectations. These anomalies occurred most frequently from midday to early afternoon, followed by late afternoon and morning (as shown in Table 1). A similar pattern was observed in an earlier set of aggression tests conducted without controlling for time, where approximately 15% of inter-site comparisons yielded unexpected low scores. Overall, our statistical analysis supports the robustness of previous studies on *L. humile* that did not account for diel variation in aggression, e.g., [46,62,65].

### 4.2. Survivorship

Overall, we were able to determine the pathogenicity and virulence of six entomopathogens isolated from *L. humile*, representing the first robust evaluation of the efficacy of organisms as potential biological control agents of Argentine ants.

Among the six evaluated strains, *Beauveria bassiana* Li053 consistently caused the highest mortality in the shortest time and was recovered from the greatest number of ants across application methods, allowing us to select it and continue testing its effects on Argentine ants (e.g., determination of LC50). Notably, in 9 out of 12 trials, this strain caused a significantly higher mortality (more than 80%) than the control treatment, and in 8 of those trials, it was the strain responsible for the highest mortality overall. In those trials, Li053 was recovered from at least 50% of all ants, and in 6 of them, it was detected in over 80% of the ants. Notably, 4 of these high-recovery cases were conducted using the immersion technique. The greater recovery from the immersion technique may likely be attributable to the higher concentration of conidia applied compared to the other application techniques.

For the other strains, the greatest mortality depended on the inoculation method and/or the colony origin. In addition, for some strains, ant mortality and strain recovery were not consistent with each other. The *Fusarium* strain Li063 caused high mortality (70%) in 5 of the trials and the highest mortality in 3 out of 12 trials but it was recovered from at most 13% of all the ants, or 18% from the cadavers, and none of those cases included the Gonnet colony nor the immersion technique. The strain *Metarhizium album* LiS/ID caused around 70% mortality in 3 trials being the highest significant mortality in 2 of them—topical method in the Bernal colony and immersion method in the RECS colony-, and was poorly recovered in the 3 cases (3% to 11% of all the ants or 3–12% from the dead ants). The strain *Aspergillus caelatus* Li015 caused more than 80% mortality in 3 out of 12 trials, but was the highest only with the topical and spray methods in the Rocha colony (its site of origin), and was recovered from 83% and 93% of total ants, respectively, or from 87% and 98% of cadavers. The strain *Purpureocillium lilacinum* Li124 caused more than 80% mortality in one trial from each colony and was the strain with the highest mortality with the spray method only in the Gonnet colony, from which it was recovered from 70% of the total ants or 78% from dead ants. *A. caelatus* Li003, although it caused more than 80% mortality in 3 trials, and it was recovered from more than 80% of all ants, it never accounted for the highest mortality in any trial. The three cases involved colonies from both reserves.

The cases of Li063 and LiS/ID could be explained by two hypotheses: either the strains were capable of killing ants but were not competitive enough to colonize cadavers due to the presence of other opportunistic organisms, such as the naturally occurring *Aspergillus* strain, or they may have weakened the ants’ immune systems, making them more susceptible to other pathogens that ultimately caused their death. A high mortality without a correspondence with a high recovery from ant cadavers was also observed when *Metarhizium anisopliae* and *Aspergillus flavus* were co-inoculated on *Acromyrmex echinator* ants [66].

Upon re-examination of the *Aspergillus* strains in ant cadavers from the control group under the immersion method, we distinguished whether the recovered fungal species matched the experimentally inoculated strains or were different. Culturing fungi from cadavers and comparing their morphology with that of the inoculated strains showed that most *Aspergillus* recovered from controls corresponded to the inoculated morphotypes, both belonging to the species *A. caelatus*. This is not surprising, given that the inoculated strains had been originally isolated from *L. humile* as part of their natural entomopathogenic fungal community. Therefore, it was not possible to differentiate naturally infected ants from those infected experimentally. For instance, in the control group from Rocha -the site with the highest *Aspergillus* prevalence-, 39% of all the ants died due to the inoculated morphotype, compared to 3% with other *Aspergillus*. Among the cadavers, 76% showed the inoculated morphotype, while only 6% had different *Aspergillus* species.

The most frequently recovered taxa in our native *L. humile* populations belonged to the *Aspergillus* genus. However, the pathogenic effects of the two *A. caelatus* strains (Li003 and Li015) varied across inoculation methods and colonies, indicating inconsistent virulence. On the contrary, strain Li053 emerged as the most virulent and consistent, inducing > 50% mortality in 11 of 12 trials and displaying an LT50 of 5 days in 64% of cases. Regarding *B. bassiana*, the natural occurrence in these ants was minimal. The apparent absence of *B. bassiana* in the field may be due to: (1) its low natural prevalence; or (2) effective ant defenses, at least for field occurrence/concentration, such as chemical secretions and grooming behaviors, limiting the host colonization. The antimicrobial effect of the pygidial gland from Argentine ants [41] and the grooming behaviors [40] have been shown to negatively affect fungi. Regardless of the cause, in this work, we were able to demonstrate that, when the strain *B. bassiana* Li053 was artificially applied to the ants, both high virulence and consistent efficacy across inoculation methods and ant colonies, as we said previously, were observed, underscoring its potential as a biological control agent against *L. humile*.

Colonies from different sites exhibited varying levels of mortality by and recovery of fungal strains, depending on the inoculation method employed. From a biological control perspective, it is desirable to use a strain that produces high mortality and high recovery. A high recovery not only matters to verify the cause of mortality but also assures high dispersal, increasing the chances of new infections. From the four sites, six fungal strains tested, and 3 inoculation methods (72 total treatments), the subsequent analysis was restricted to only those cases that met the following criteria: the strain caused 50% of mortality or more, and the same strain was recovered from at least 50% of the cadavers (from now on referred to high-mortality-high-recovery). Based on these criteria, we inferred that the fewer strains capable of causing high-mortality-high-recovery, the more resistant the colony was considered to be. Therefore, 20 high-mortality-high-recovery cases were recorded out of 72. Bernal colony appeared to be the most susceptible to the inoculated strains, showing 7 out of those 20 cases, whereas RECS showed 5 cases, and both Rocha and Gonnet each showed only 4 cases. In addition, the colony exhibiting the lowest mortality in the control group was from the urban site of Gonnet. This low mortality (25% on average) might be attributed to a high resistance and/or a reduced natural pathogen load carried by these ants. Control ants from Gonnet consistently exhibited the lowest pathogen loads, ranging from 5 to 18% among all ants. Colony-level variation effects in pathogen susceptibility have been documented in other ant species [67,68]; however, most studies to date have relied on limited colony sampling or lacked true replication (for a detailed discussion, see [45]).

Using the same criteria (high mortality and high recovery), 8 out of 20 qualifying cases involved the spray method, while the topical and immersion techniques accounted for 6 cases each. These findings suggest no substantial differences in effectiveness among inoculation methods, at least in the cases that met the selected criteria. However, when fungal strain identity is considered, four strains met the criteria, with Li053 being the most frequently associated with high mortality and recovery (9 cases), followed by Li003 (5 cases), Li015 (4 cases), and Li124 (2 cases). All four strains were associated, at least once, with both the topical and spray methods. In contrast, only Li053 and Li003 were effective when applied via immersion. These results suggest that the immersion method may provide a more consistent assessment of fungal efficacy, whereas the spray and topical methods yielded more variable outcomes. The greater consistency observed with immersion might be due to the higher conidial load and uniform coverage achieved through this method, which could have outcompeted or suppressed the naturally occurring pathogens carried by the ants. Further research is needed to clarify the underlying mechanisms.

Despite the lower conidial load associated with topical inoculation, this method induced mortality up to 70% in some cases (e.g., strains Li015 in the Rocha colony, and Li053, Li063, Li124, LiS/ID in the Bernal colony). This result suggests that infection success may not depend strictly on the number of conidia present on the insect cuticle, but rather on the successful germination and internal colonization by a small number of conidia. Even a relatively low conidial concentration, such as the LC50 (1.2 × 10^6^ conidia/mL), was sufficient to induce substantial mortality. Although higher concentrations led to more pronounced mortality levels (Figure 5), the LC90 (2.1 × 10^9^ conidia/mL) was an order of magnitude greater than the concentration used in the virulence assays. This suggests that the concentrations applied during the immersion treatments with *B. bassiana* Li053 may have been insufficient to achieve complete (100%) mortality. We also found that the increasing conidial concentration was associated with higher mortality and a greater proportion of fungal recovery. Thus, conidial concentration appears to influence the speed and extent of mortality, but infection can occur even at relatively low concentrations if conditions for germination and host colonization are favorable. Notably, as the concentration of Li053 increased, the recovery of the naturally occurring *Aspergillus* species decreased, while the recovery of Li053 increased. This suggests that higher concentrations of the inoculated strain may enhance its competitive ability against naturally occurring fungi.

*B. bassiana* is a generalist entomopathogenic fungus, although most isolates change their effectiveness depending on the host [69]. Field studies have shown no adverse effects on vertebrates, beneficial insects, earthworms, or plants ([70], as cited [71]). Additionally, *B. bassiana*-based biocontrol products have undergone rigorous safety assessments and have been approved for use in multiple countries. As with many microorganisms, *B. bassiana* may cause allergic reactions in individuals with high conidial exposure; however, such risks can be mitigated through appropriate protective measures and proper handling and application protocols [71,72].

In summary, this study represents the first comprehensive investigation into the use of natural enemies of *L. humile* as potential biological control agents. Furthermore, it is among the few studies that assess the effects of fungal strains already harbored by target ant populations [45]. We identified one strain, *B. bassiana* Li053, that consistently induced high mortality across multiple colonies and inoculation methods, indicating its potential as a candidate for the biological control of Argentine ants. Field experiments are required to determine whether this potential is maintained under natural conditions.

## Figures and Tables

**Table 1:** Frequency of scores obtained from aggression tests performed between pairs of ants coming from the same site (CT) or from different sites (vs.) discriminated by time (morning, midday to early afternoon, and late afternoon).

Sites/Time	Morning (9:00 to 12:00)					Midday to Early Afternoon (12:00 to 17:00)					Late Afternoon (17:00 Onwards)				
Score	0	1	2	3	4	0	1	2	3	4	0	1	2	3	4
Rocha CT		3				2	14				1	7			
RECS CT		3					7					5			
GONNET CT		6					7					6			1
BERNAL CT		6					7					7			
RECS vs. ROCHA		2	1		3			1	1	4				2	4
GONNET vs. BERNAL			1		5	1	1			4		1	2	2	1
ROCHA vs. BERNAL			1		10					7					13
RECS vs. BERNAL					6			1		5			2	1	3
RECS vs. GONNET		1		1	4	2	2		1	3				2	4
GONNET vs. ROCHA				2	4					6		2			7

**Table 2:** Molecular identification of fungal strains isolated from *Linepithema humile* based on BLAST searches of 28S rDNA sequences. For each strain, the species showing the highest sequence identity (%) is indicated, based on comparison with sequences from reference strains deposited in recognized culture collections available in GenBank. Species-level identifications were confirmed when both sequence identity and morphological features were consistent. Codes from culture collections are as follows: ARSEF—ARS Collection of Entomopathogenic Fungi (USDA, Ithaca, NY, USA); ATCC—American Type Culture Collection (Manassas, VA, USA); CBS—Westerdijk Fungal Biodiversity Institute (Utrecht, The Netherlands).

Strain	Sampling Site	28S rDNA BLAST Result—Species and % Identity	Final Species Identification
Li053	ROCHA	*Beauveria bassiana* ATCC 26854 and *B. bassiana* CBS:212.61 (99.68%)	*Beauveria bassiana*
LiS/ID	ROCHA	*Metarhizium anisopliae* CBS:662.67 (97.96%) and *M. album* ARSEF 2082 (92%)	*Metarhizium album*
Li124	ROCHA	*Purpureocillium lilacinum* CBS:129410 and *P. lavendulum* CBS:128678 (100%), and *P. lilacinum* ATCC 10114 (99.29%)	*Purpureocillium lilacinum*
Li063	ROCHA	*Fusarium incarnatum* CBS:173.30, *F. oxysporum* CBS:130308, *F. oxysporum* f. *cubense* strain ATCC 96285 and *F. redolens* CBS:128.73 (100%)	*Fusarium oxysporum*
Li003	RECS	*Aspergillus caelatus* CBS:763.97 (99.75%)	*Aspergillus caelatus*
Li015	ROCHA	*Aspergillus caelatus* CBS:763.97 (94.84%)	*Aspergillus caelatus*

## Data Availability

In addition to the data provided in the Supplementary Material, other raw data supporting the conclusions of this article will be made available by the authors upon reasonable request.

## Supplementary Materials

The following supporting information can be downloaded at: https://www.mdpi.com/article/10.3390/insects16070677/s1, Table S1: Raw survival data used for statistical analyses. The file is provided in Excel format and contains separate sheets for each study site and inoculation method. Within each sheet, data are organized by individual ants and include: time of death (in days), censoring status (0 = dead, 1 = censored), and treatment (fungal strain). The dataset is formatted according to the input requirements of SYSTAT software.
